# Associations of Cardiovascular and All-Cause Mortality with Metabolic Syndrome in Hemodialysis Patients: A Prospective Single-Center Study

**DOI:** 10.3390/medicina55100694

**Published:** 2019-10-16

**Authors:** Zorica Dimitrijevic, Andriana Jovanovic, Mina Cvetkovic, Tamara Vrecic, Emina Kostic, Branka Mitic

**Affiliations:** 1 Faculty of Medicine, University of Niš, 18000 Niš, Serbia; kosticemina2@yahoo.com (E.K.); dmmitic@ptt.rs (B.M.); 2 Clinic for Nephrology, Clinical Center Niš, 18000 Niš, Serbia; anchej89@gmail.com (A.J.); mina_cvetkovic@yahoo.com (M.C.); tamaravrecic@live.com (T.V.)

**Keywords:** metabolic syndrome, hemodialysis, cardiovascular, mortality

## Abstract

*Background and objectives:* Metabolic syndrome (MetS) is a cluster of risk factors, such as abdominal obesity, insulin resistance, dyslipidemia and hypertension, that together increase the risk of cardiovascular disease. Chronic hemodialysis (HD) patients have multiple comorbidities and many metabolic disorders, causing the frequent occurrence of metabolic syndrome. The goal of this study was to assess the prevalence of MetS in HD patients, and its association with all-cause and cardiovascular (CV) mortality. *Patients and methods*: A total of 138 HD patients were included in this prospective study. We analyzed demographic, anthropometric and biochemical data. Outcome measures were all-cause and CV mortality during the three-year follow-up. *Results*: MetS was diagnosed in 57.24% of enrolled patients. During the 36 months of follow-up, 33 patients died. MetS patients showed a significantly higher mortality rate than non-MetS (30.4% versus 16.36%, *p* < 0.001). The association of different MetS components with cardiovascular mortality reached significance when a minimum of three components were present (1.81 (95% confidence interval CI = 1.21–2.33)), with a grouped increase in effect size for subjects with four or five MetS components. Subjects with MetS exhibited nearly twice as high risk for all-cause (hazard ratio HR = 1.99 (95%CI) = 1.42–2.97) and 2.5 times for CV (HR = 2.51 (95%CI) = 1.25–3.83) mortality compared with those without MetS, after adjustment for age, gender, and cardiovascular disease. *Conclusions:* The study demonstrates that MetS is widespread in HD patients. In future, the focus must be on an active screening approach, and treatment of cardiometabolic risk factors, aiming to reduce mortality.

## 1. Introduction

Metabolic syndrome (MetS) is defined as a cluster of complementary physiological, biochemical, metabolic, and clinical determinants, that increase the risk of cardiovascular disease, diabetes, and all-cause mortality [1]. Most studies confirm that MetS is associated with a near doubling of cardiovascular disease (CVD) risk, and a 5-fold increased risk for incident type 2 diabetes mellitus (DM) [2], in addition to great economic healthcare costs. The MetS prevalence escalates into epidemic proportions in the urbanized world and developing countries, and increases with age, region, and population; however, prevalence varies widely depending on the definition used for treatment strategies. Various definitions of MetS have been introduced by the National Cholesterol Education Program’s Adult Treatment Panel III (NCEP-ATP III) [3], the International Diabetes Federation (IDF) [4], and the World Health Organization (WHO) [5]. According to NCEP-ATP III, MetS is defined as the presence of at least three of the possible five abnormalities: a waist circumference of >102 cm for men, >88 cm for women; fasting glucose value of >5.6 mmol/L; blood pressure of >135/85 mmHg; serum triglycerides levels of >1.7 mmol/L; serum high-density cholesterol (HDL-C) levels of <1.03 for men, <1.29 mmol/L for women.

Metabolic syndrome is frequently observed in patients with chronic kidney disease (CKD) and is associated with the progression of CKD [6]. Hypertension and hyperglycemia are components of metabolic syndrome that play an important role in renal function deterioration. The prevalence of metabolic syndrome in patients treated with chronic hemodialysis (HD) is exceptionally high, ranging between 30% and 70% [7,8,9]. Namely, patients undergoing chronic HD treatment have multiple comorbidities and metabolic disorders, which explains the frequent occurrence of metabolic syndrome in this population. On the other hand, metabolic syndrome itself increases the risk of cardiovascular diseases, and therefore causes a vicious cycle. It is generally known that cardiovascular disease is the leading cause of death across all age groups of patients with end-stage renal disease (ESRD) [9]. However, there is limited information on mortality rates in patients on HD with MetS. A recent meta-analysis of eight studies with a total of 1273 ESRD patients demonstrated that patients with MetS have a higher risk of all-cause mortality compared to patients without metabolic syndrome [10]. However, these studies comprised mostly of Asian HD patients [11,12,13] and patients on peritoneal dialysis [14,15,16]. Only two prospective studies [17,18] investigated mortality in Caucasian HD patients with MetS. Therefore, the purpose of our study was to investigate the prevalence of metabolic syndrome in patients on chronic HD, and to explore the risk of overall and cardiovascular (CV) mortality.

## 2. Materials and Methods

This prospective cohort study was carried out at the Nephrology Clinic of the Clinical Center Niš, Serbia. The study was approved by the Ethics Committee of Faculty of Medicine, University of Niš, Serbia (date: 15 May 2015; approval number: 12-3627-2/1), and all the participants provided written informed consent. The “STrengthening the Reporting of OBservational studies in Epidemiology” (STROBE) statement was used for reporting the study.

We recruited 168 patients older than 18 years, who had received chronic HD treatment for at least the past three months. Patients with inadequate HD therapy, with a mean dialysis parameter (Kt/V) <1.2, patients with infection during the last three months, history of malignancy, unwillingness to have their waist circumference (WC) measured, and patients with incomplete clinical and laboratory data, were excluded.

Baseline data, including demographic characteristics, dialysis vintage, Kt/V, anthropometric measurements (body height, body weight, waist circumference), biochemical analyses, as well as data on selected comorbidities, were obtained. Clinical identification of patients with MetS features was based on the criteria suggested by the NCEP-ATPIII (National Cholesterol Education Program Adult Treatment Panel III) [3]:

1. Abdominal (visceral) obesity: waist circumference ≥102 cm in men and 88 cm in women;
2. Fasting hyperglycemia ≥5.6 mmol/L or using diabetes medications;
3. Fasting triglycerides ≥1.7 mmol/L or taking triglyceride-lowering agents;
4. High-density lipoprotein cholesterol (HDL-C) <1.03 mmol/L for men and <1.29 for women or taking cholesterol-lowering agents; and
5. Hypertension (systolic blood pressure ≥130, diastolic blood pressure ≥85) or using antihypertensive medication.

MetS was defined by the presence of three or more of the cardiometabolic components listed above, as compared to individuals without MetS (i.e., with two or less cardiometabolic components).

Outcome measures were all-cause and cardiovascular mortality during the three-year follow-up. Cardiovascular mortality was defined as sudden death, myocardial infarction, angina, congestive heart failure, malignant arrhythmia, and stroke.

### Statistical Analysis

Continuous variables were presented as mean ± standard deviation (SD), and categorical variables were presented as number (N) or percentage (%). The Student’s t-test was used to compare two groups of data if there was a normal distribution of frequencies within the group. Differences in the categorical variables were tested with a chi-square test. Univariate logistic regression and multivariable logistic regression analyses were performed to predict potential significant predictors of metabolic syndrome, employing the backward elimination modeling technique. To evaluate the association between each MetS component, their grouping, and the MetS, with all-cause and cardiovascular mortality, we utilized multivariable Cox proportional hazard models. In all models, age, gender, and cardiovascular disease were treated as covariates, and adjusted for in Cox multivariable models. When comparing each of the MetS components (i.e., abdominal obesity, glucose intolerance, hypertension, low HDL-C, and hypertriglyceridemia) with mortality, all five components were included in the same regression model, to adjust for each other as well as for the aforementioned covariates. Receiver operating characteristic (ROC) curve analysis was carried out to evaluate the predictions’ accuracy.

## 3. Results

**Figure 1:**
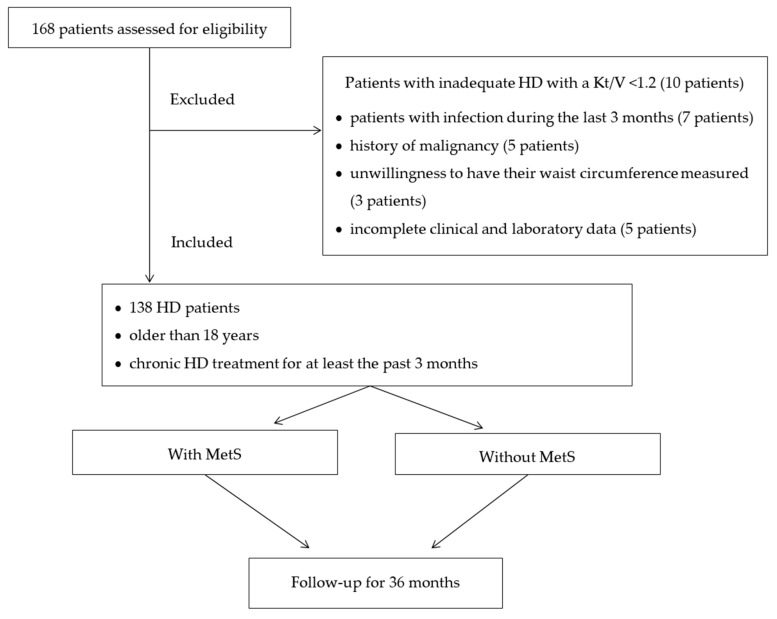
The flowchart of the study design.

Ten patients were excluded because of low KT/V, seven because of infection, five had malignancy, three were excluded because of their unwillingness to have their WC measured, and for three patients there were incomplete clinical and laboratory data. The final study population included 138 stable patients. Figure 1 shows the flowchart of the study design.

The mean age of the patients was 65.27 ± 11.5 years, 59.4% of patients were male, dry weight was 65.3 ± 13.7 kg, and dialysis vintage 67.74 ± 53.09 (range 15.2–251.4) months. The baseline characteristics of the participants are summarized in Table 1.

Out of the total number of HD patients, 71 (51.44%) had a body mass index (BMI) which was above optimal, i.e., 49 (35.5%) of the patients were overweight, and 22 of them (15.94%) were obese. Eight percent of the patients were malnourished.

The patients were divided into two groups according to the presence or absence of the metabolic syndrome, according to the above-listed NCEP: ATP III criteria. The prevalence of MetS in our study group was 57.24%.

The most frequent component of MetS was hypertension, diagnosed in 72 patients (91.14%), followed by increased waist circumference (83.54%) (Table 2).

In the MetS group, the mean age of the participants was 56.2 ± 12.4 years, and 51.8% of the participants were female. In the non-MetS group, the mean age was 49.1 ± 15.3 years, and 48.2% were females. Systolic blood pressure, waist circumference, BMI, serum levels of triglycerides and CRP were significantly higher in the MetS group than in the non-MetS group (all, *p* < 0.001), in addition to glucose, total cholesterol, and low-density cholesterol (LDL-C) (all, *p* < 0.05). However, the HDL-C levels were significantly lower in the MetS group than in the non-MetS group (*p* < 0.05). Furthermore, incidences of hypertension and diabetes mellitus were higher in the MetS group than in the non-MetS group (*p* < 0.05 and *p* < 0.001, respectively).

**Figure 2:**
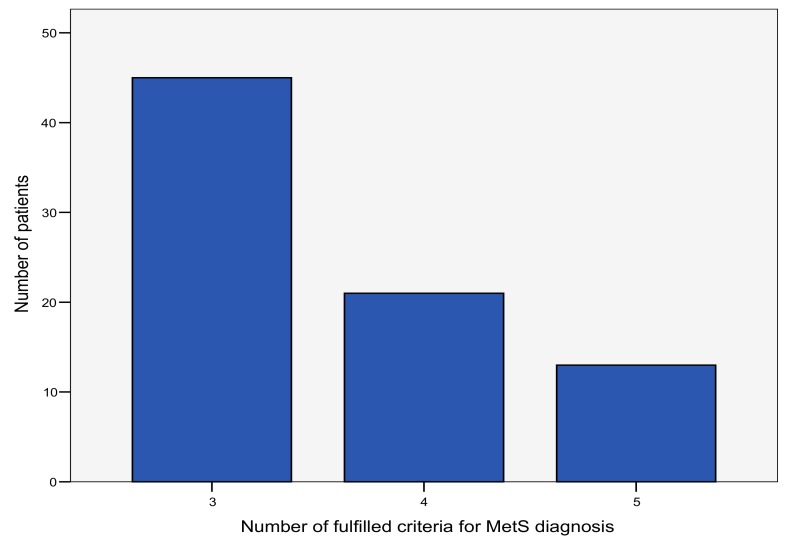
The number of fulfilled criteria for the diagnosis of metabolic syndrome (MetS) in the study population.

A majority of patients, 45 (56.96%), fulfilled the three diagnostic criteria for metabolic syndrome, while 13 patients (16.45%) met all five criteria (Figure 2).

Female patients have a 1.7-fold greater risk of metabolic syndrome (OR (95% CI) = 1.66 (1.23–2.14); *p* < 0.001). Age is also associated with metabolic syndrome among HD patients, and patients aged above 45 years were almost five times more exposed to the risk of developing metabolic syndrome (OR (95% CI) = 4.92 (3.23–9.18); *p* = 0.021). Obese individuals have a nine times higher risk of metabolic syndrome, compared to those of normal weight. Finally, higher CRP is also associated with the risk of MetS (Table 3).

During the 36 months of follow-up, 33 patients died. Overall, those with MetS showed a significantly higher mortality rate than those without MetS (30.4% versus 16.36%; *p* < 0.001). The cardiovascular mortality rate among MetS patients was 66.6%, compared to 44.4% in the non-MetS group (*p* < 0.05).

The association of individual cardiometabolic component with all-cause and CV mortality, while controlling for confounding variables, is presented in Table 4. The multivariable-adjusted risk of cardiovascular mortality associated with hypertension (hazard ratio, HR = 1.58 (95% confidence interval CI = 1.24–1.94)) and abdominal obesity (HR = 1.52 (95% CI = 1.13–1.84)) was statistically significant. The association of the different MetS components with cardiovascular mortality reached significance when a minimum of three components were present (1.81 (95% CI = 1.21–2.33)), with a grouped increase in effect size for subjects with four or five MetS components (HR = 1.90 (95% CI = 1.19–2.99) and HR = 2.88 (95% CI = 1.76–4.17), respectively). The model for these MetS components and their clustering was comparable to all-cause mortality, regardless of diminished statistical significance (the HRs (95% CI) for components three, four, and five were 1.37 (0.88–2.59), 1.62 (0.90–3.01), and 1.81 (0.76–4.31), respectively).

Actually, as displayed in Table 4, MetS patients exhibited nearly twice the risk of all-cause (HR = 1.99 (95% CI) =1.42–2.97), and 2.5 times the risk for CV (HR = 2.51 (95% CI) = 1.25–3.83) mortality, compared to those without MetS, after age, gender, and CVD adjustment.

**Figure 3:**
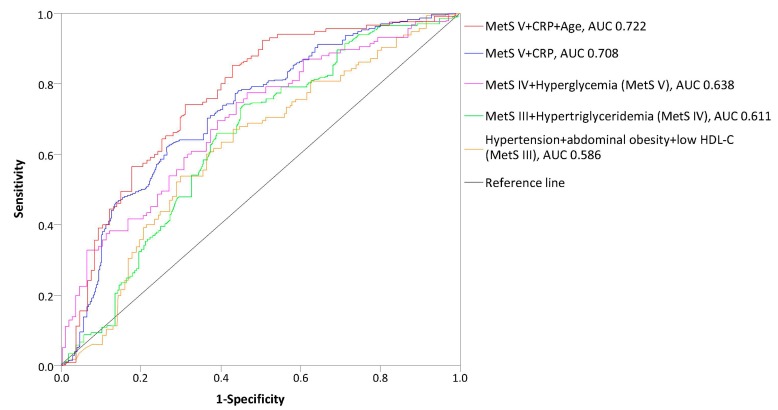
Receiver operating curve (ROC) for mortality risk score prediction according to MetS components in HD patients; AUC, area under the curve.

Finally, the risk of mortality was predicted based on the number of Mets components. The AUC of the MetS risk score in predicting cardiovascular mortality, as analyzed using the ROC curve, was 0.638 for the total MetS score, including all five Mets components (Figure 3). However, when CRP and age were included in predictions, the sensitivity of the mortality prediction increased from 66.1% to 76.4%, and the AUC increased from 0.638 to 0.722 (*p* < 0.001).

## 4. Discussion

Our study demonstrated the presence of metabolic syndrome in 57.24% of patients undergoing chronic HD treatment at the Clinical Center Nis, Serbia, which is in accordance with the results of several other studies [19,20]. However, Young and coworkers reported an even higher prevalence of MetS (up to 70%) in the HD population, notably among diabetic, female, and white patients [21]. Data about the frequency of metabolic syndrome among HD patients are still conflicting, and the prevalence of MetS may vary depending on the definition used. In the study of Vogt et al. [17], prevalence of MetS in chronic HD patients ranged from 51% to 74.5%, based on the criteria used for MetS diagnosis. Disparity, as mentioned above, could also be explained by differences between sample size, race, dietary habits, and culture. This indicates that we still lack a unique definition that reflects the real spectrum of the MetS epidemiology.

Hypertension is probably a fundamental component of MetS, with almost 85% of MetS patients having this condition [22]. As expected, the most common MetS criteria observed in our patients was hypertension. As many as 91.14% of HD patients with MetS suffered from hypertension, and this percentage is significantly higher, compared to other studies [23,24]. Hypertension in our MetS patients is apparently volume-dependent, because we found statistical significance in interdialytic weight gain among these patient groups (*p* < 0.001).

Waist circumference is an important component of MetS. Roughly 83.54% of the MetS patients had an increased waist circumference, which directly expresses the proportion of abdominal fat and central obesity. Due to heightened abdominal adiposity and insulin resistance, MetS is thought to be correlated with a chronic low-grade inflammatory response, which is characterized by overt cytokine production and inflammatory signaling pathways [25]. It has been shown that, despite the presence of MetS in an individual patient, CRP levels were an independent predictor of future CV events [26] and unfavorable outcomes of MetS [27]. Similar to Song et al. study [28], we also proved that CRP level plays a significant role in the development of MetS.

As the DOPPS study clearly demonstrated that CRP influences mortality in HD patients [29], we may need to be aware of inflammatory markers and control low-grade inflammation. This may be beneficial in the prevention and management of metabolic disorders.

We further found that elderly subjects were at a higher risk of metabolic syndrome, which is compatible with the results of several authors [24,30,31]. However, the study of Mozaffarian et al., carried out on 4258 US adults 65 years or older, suggested a limited utility of the MetS concept for predicting total, CV, or non-CV mortality in older men and women, compared with the assessment of fasting glucose level and blood pressure alone [32].

Sex differences in the prevalence of metabolic syndrome have been observed in several studies. We demonstrated that female subjects were 1.7 times more exposed to the risk of developing metabolic syndrome. The female gender dominance seen in our data is similar to several other studies [31,33,34]. An increase in the prevalence of metabolic syndrome in women is mainly due to the persistent rise in obesity in women, with approximately two million more women than men affected in the United States [35], as well as in developing countries, including Asian countries [20,36].

The clinical utility of MetS is based on its prediction of the clinical outcome, especially regarding all-cause and cardiovascular mortality. Pérez de José et al. [37] analyzed the effects of MetS on cardiovascular events in 100 HD patients and found no difference in mortality between patients with or without MetS; however, they reported a higher hospitalization rate due to all causes in patients with MetS. Earlier investigations [38,39,40] pointed out that MetS increases the risk of mortality from CV and other diseases in the general population, but it is not yet fully clarified to what degree the existence of metabolic syndrome influences the mortality of HD patients. Our study demonstrated that HD patients have an increased risk of all-cause and CV mortality associated with MetS. Importantly, this effect alteration was driven by the blood pressure, visceral obesity and, to a lesser extent, by low HDL-C components of MetS.

To conclude, according to the results of this study, it is evident that the presence of metabolic syndrome can lead to the occurrence of cardiovascular death and a shorter life expectancy in Caucasian patients treated with HD, and the risk of CV death increases significantly with an increased number of MetS components. Patients with identified MetS have almost two times higher statistically significant probability for CV mortality than individuals without MetS. Accordingly, it is important to notice any metabolic changes in these patients and thoroughly approach treatment, in order to prevent life-threatening consequences and therefore prolong the patient’s life.

Our study has limitations. Firstly, the study recruited patients from only one HD center, and we collected baseline information at a single time point. Secondly, we did not reevaluate the MetS status at the end of the follow-up, so that new onset MetS was not diagnosed, which could affect the results of the research.

## 5. Conclusions

In summary, our study provides evidence of the high prevalence of metabolic syndrome in HD patients. In future, the focus must be on an active screening approach, assessment, and treatment of cardiometabolic risk factors. Targeting hemodialysis patients with metabolic syndrome is critical in reducing the incidence of cardiovascular and all-cause mortality.

## Figures and Tables

**Table 1:** Demographic, hemodynamic, anthropometric and biochemical characteristics of the subjects.

	At Baseline
Age (years)	64.78 ±11.33
Men (%)	82 (59.4%)
Dialysis vintage (months)	67.74 ± 53.09
Arteriovenous fistula (%)	83.3
Kt/V	1.36 ± 0.70
Dry weight (kg)	65.3 ± 13.7
Interdialtic weight gain (kg)	2.6 ± 2.1
Body Mass Index (kg/m^2^)	24.6 ± 3.9
Waist circumference (cm)	94.1 ± 13.1
Systolic blood pressure (mmHg)	134.4 ± 31.6
Diastolic blood pressure (mmHg)	92.2 ± 11.3
Hypertension (%)	103 (73.91%)
Diabetes mellitus (%)	31 (22.46%)
Hyperlipidemia (%)	44 (31.88%)
Hgb (g/L)	104.22 ± 5.10
Cholesterol (mmol/L)	5.1 ± 1.2
Triglycerides (mmol/L)	2.9 ± 1.9
LDL-C (mmol/L)	3.1 ± 0.5
HDL-C (mmol/L)	1.3 ± 0.7
s. Albumins (g/L)	33.00 ± 4.89
Total protein (g/L)	69.91 ± 7.87
s. Creatinine (μmol/L)	842.94 ± 189.38
CRP (mg/L)	3.94 ± 2.84
PTH (pg/mL)	339.34 ± 225.97

Data are expressed as mean (± standard deviation); Hgb = hemoglobin; LDL-C = low density cholesterol; HDL-C = high density cholesterol; CRP = C-reactive protein; PTH = parathormone.

**Table 2:** Baseline characteristics of the patients with or without metabolic syndrome.

	Number of Patients		
Metabolic Syndrome (n, %)	Yes 79 (57.24%)	No 59 (42.76%)	*p* Value *
ATP III MS Criteria			
Hypertension	72 (91.14%)	50 (84.7%)	<0.05
Increased waist circumference	66 (83.54%)	20 (33.89%)	<0.05
Low HDL-C	61 (77.21%)	19 (32.20%)	<0.001
High triglycerides	52 (65.82%)	8 (13.55%)	<0.001
Hyperglycemia	36 (45.57%)	11 (18.64%)	<0.001
Age (years)	56.2 ± 12.4	49.1 ± 15.3	0.022
Gender (%)			
Men	27.4	72.6	<0.001
Women	51.8	48.2	0.71
Dialysis vintage (months)	52.3 ± 44.6	69.3 ± 42.7	<0.05
Dry weight (kg)	71 ± 29	56 ± 18.2	<0.001
Interdialytic weight gain (kg)	3.2 ± 1.3	2.2 ± 0.9	<0.001
Body Mass Index (kg/m^2^)	29.1 ± 2.18	23.1 ± 1.8	<0.001
Waist circumference (cm)	108.3 ± 12.6	75.2 ± 14.3	<0.001
Obesity	27 (34.17%)	11 (13.92%)	<0.05
Diastolic blood pressure (mmHg)	83.2 ± 9.2	72.3 ± 11.4	<0.05
Hgb (g/L)	101.2 ± 7.6	103.1 ± 4.2	0.66
Blood glucose level (mmol/L)	10.6 ± 4.3	6.2 ± 2.8	<0.05
Cholesterol (mmol/L)	4.64 ± 1.13	3.36 ± 1.86	<0.05
Triglycerides (mmol/L)	2.96 ± 1.3	1.88 ± 0.96	<0.001
LDL-C (mmol/L)	3.14 ± 1.7	2.16 ± 1.8	<0.05
HDL-C (mmol/L)	0.79 ± 0.56	1.02 ± 0.3	<0.05
s. Albumin (g/L)	34.2 ± 4.8	35.3 ±8.3	0.82
Total protein (g/L)	72.2 ± 4.6	71.3 ± 6.2	0.44
s. Creatinine (μmol/L)	726.2 ± 108.3	699.5 ± 159.5	0.39
CRP (mg/L)	7.01 ± 2.8	2.3 ± 2.1	<0.001
PTH (pg/mL)	319.86 ± 101.3	298.2 ± 179.3	0.18

* Student’s *t*-test.

**Table 3:** Multivariate logistic regression method showing significant predictors of MetS in the study population.

	Metabolic Syndrome		OR (CI 95%)	*p* Value
	Yes	No		
**Gender (%)**				<0.001
Male	27.4	72.6	reference	
Female	51.8	48.2	1.66 (1.23–2.14)	
**Age (%)**				0.021
<45 years	12.2	87.8	reference	
>45years	35.3	64.7	4.92 (3.23–9.18)	
**Body Mass Index (%)**				<0.001
<20	18.2	81.8	0.38	
21–25	41.1	58.9	reference	
26–30	73.5	26.5	4.22 (2.3–7.14)	
>30	81.8	18.2	8.91 (6.34–10.2)	
**CRP (mg/L) (%)**				0.038
<5	20.1	79.9	reference	
5–10	38.6	61.4	1.71 (1.13–3.19)	

**Table 4:** Association of the individual MetS component, MetS components grouping, and MetS with the all-cause and cardiovascular mortality after two years of follow-up.

Number of Deaths	Cardiovascular Mortality		All-Cause Mortality	
	24 (72.7)		9 (27.3)	
	HR (95%)	*p* Value	HR (95%)	*p* Value
**MetS components**				
Hypertension	1.58 (1.24–1.94) ^a^	<0.001	1.70 (1.24–2.76) ^a^	0.036
Abdominal obesity	1.52 (1.13–1.84) ^a^	<0.001	1.12 (0.77–1.81) ^a^	0.28
Low HDL-C	0.69 (0.56–1.09) ^a^	0.033	1.17 (0.75–1.66) ^a^	0.053
Hypertriglyceridemia	1.15 (0.82-1.53)	0.08	1.21 (0.77–1.63) ^a^	0.49
Hyperglycemia	0.89 (0.56–1.42) ^a^	0.23	1.19 (0.70–1.75) ^a^	0.55
**Number of MetS components**				
0	1.00	0	1.00	0
1	0.88 (0.58–1.46)	0.75	0.60 (0.33–1.07)	0.21
2	0.94 (0.70–1.55)	0.44	0.60 (0.41–1.22)	0.33
3	1.81 (1.21–2.33)	<0.001	1.37 (0.88–2.59)	0.66
4	1.90 (1.19–2.99)	0.05	1.62 (0.90–3.01)	0.38
5	2.88 (1.76–4.17)	<0.001	1.81 (0.76–4.31)	0.19
No (<3 components)	1.00			
Yes (≥3 components)	2.51 (1.25–3.83) ^b^	<0.001	1.99 (1.42–2.97) ^b^	<0.001

HR = hazard ratio; MetS = metabolic syndrome; HDL-C = high density cholesterol; ^a^ Hazard ratios (95% CIs) adjusted for each other in addition to age, gender, and previous cardiovascular disease; ^b^ Hazard ratios (95% CIs) adjusted for age, age, gender, and previous cardiovascular disease.
